# Exploring big data-driven innovation in the manufacturing sector: evidence from UK firms

**DOI:** 10.1007/s10479-021-04077-1

**Published:** 2021-04-21

**Authors:** Mujahid Mohiuddin Babu, Mahfuzur Rahman, Ashraful Alam, Bidit Lal Dey

**Affiliations:** 1 Coventry University, Coventry, UK; 2 University of Lincoln, Lincoln, UK; 3 Salford University, Manchester, UK; 4 Brunel University, London, UK

**Keywords:** Big data analytics, Data-driven innovation (DDI), Data products, Data governance

## Abstract

Although innovation from analytics is surging in the manufacturing sector, the understanding of the data-driven innovation (DDI) process remains a challenge. Drawing on a systematic literature review, thematic analysis and qualitative interview findings, this study presents a seven-step process to understand DDI in the context of the UK manufacturing sector. The findings discuss the significance of critical seven-step in DDI, ranging from conceptualisation to commercialisation of innovative data products. The results reveal that the steps in DDI are sequential, but they are all interlinked. The proposed seven-step DDI process with solid evidence from the UK manufacturing and research implications based on dynamic capability theory, institutional theory and TOE framework establish the building blocks for future studies and industry practice.

## Introduction

The impact of big data analytics (BDA) on organizational outcomes, particularly in manufacturing and service contexts, has received incessant attention from both academia and practice. Different complementary trends and digital technologies, such as the diffusion of smart devices, social network platforms, the improvement and diffusion of cloud computing and Internet of Things have been considered relevant to enable and enhance the BDA phenomenon (e.g. Dumbill, 2013). In the current day business environment, BDA has been regarded as the fastest emerging momentum which has implications for various organizational opportunities such as, business intelligence and cognitive computing, business innovation and overall performance (Akter et al., 2020; Davenport & Kudyba, 2016; Delen & Demirkan, 2013; Dubey et al., 2019; Moktadir et al., 2019).

The manufacturing literature broadly conceptualizes BDA as the organization’s analytical capability to collect, process, analyze and interpret large datasets to extract out insights relevant for effective decision making and operational performance (Akter et al., 2016; Minelli et al., 2013; Moktadir et al., 2019; Wang et al., 2018; Wang & Hajli, 2017). The traditional manufacturing industries are now transformed by the application of data-driven revolution and BDA. As a result, new manufacturing industries have better efficiency and accuracy that can have a positive impact across the entire organisation in terms of process and product innovation and operational efficiency (O’Donovan, 2015). Because of the exponential advances in information and communication technology (ICT) such as artificial intelligence, blockchain, cloud computing and the internet of things (IoT), manufacturing industry is inundated with vast amount of global data (Waller & Fawcett, 2013; Wang et al., 2016). Such a big amount of data enhances the manufacturing organization’s capability not only to make informed decisions, alleviate risks, improve operational procedures but also to facilitate data-driven innovation such as, conducting market analyses for particular products, introducing new products or upgrading existing product lines (Dubey et al., 2016, 2019; Moktadir et al., 2019; Schoenherr & Speier-Pero, 2015; Zhong et al., 2016).

Over the last decade, due to a more pervasive and ubiquitous diffusion of digital technologies, digital innovation has now entered its golden age (Fichman et al., 2014). BDA, Internet of Things and other technological developments allow companies to gather a considerable amount and variety of data which would trigger a meaningful direction to innovate within a company and have a significant impact on innovation management (Trabucchi & Buganza, 2019; Trabucchi et al., 2018). In the manufacturing sector, data is abundant in large volume due to the diffusion of smart technologies, devices, and applications. Moreover, the growth and ease of operations of cloud computing have enabled the organizations to gain access, store, retrieve big data. Furthermore, manufacturers can have large application, conditional and usage data from their produced smart products through IoT which converges different technology, objects, integrated technology, and semantic approach. Such a big amount of data and computational power have enabled the organizations to facilitate data-driven innovation. Despite the enormous growth of big data research in academia and practice, exploiting BDA for enhancing innovation in the manufacturing sector is still unexplored. Indeed, *the extant literature has reported a dramatic decline of* competitive advantage with big data analytics in recent years due to its lack of innovation, value or productivity. Specifically, little is known about the effective utilisation of big data for developing data products in manufacturing. Consequently, this study is an initial attempt towards bridging this knowledge gap identified in the literature. More precisely, drawing on the recent research agenda, this study examines the following research question: what is the data-driven innovation process in the manufacturing sector?

To answer the above mentioned research question, the paper presents a framework to understand the data-driven innovation process drawing on a systematic literature review, thematic analysis and qualitative interviews (n = 23). We propose a seven-step process with examples in the manufacturing sector in the UK. The UK manufacturing sector plays a very significant role which is appreciated by the government particularly for the contribution to innovation and exports (Xu et al., 2018). During the Covid-19 Pandemic, many UK manufacturers renovated their factories to support ongoing health system reforms. The UK government is now more committed to enhance the capacity of the manufacturing sector by incorporating the other sectors through inter-industry linkages and the employment of highly skilled labour. There are four major sections in the paper. First, we present the theoretical framework for data-driven innovation using dynamic capability theory, institutional theory and technology–organisation–innovation theory. Second, we discuss the methodological foundations based on a systematic review, thematic analysis and in-depth interviews. Finally, we present the findings of data-driven innovation with a future research agenda.

Our study offers several contributions. First, this study applies the concept of BDA in the manufacturing sector. Manufacturing firms encompass multiple processes in developing a product, thereby they offer a distinct setting to analyze the application of BDA. Our paper finds that BDA can be utilized for enhancing innovation in the manufacturing sector. Second, we offer a theoretical contribution. We apply a multi-theoretical approach wherein DCT, institutional theory and TOE framework are utilized to understand DDI in manufacturing firms. Our findings suggest that organization’s capabilities and stakeholder factors play a part in DDI, thereby contributing to DCT and institutional theory respectively. Further, this study supports the view that the process, consisting of technology, organization and environment, acts as a factor in DDI, which contributes to the TOE framework. Third, this study provides deeper insights into the seven-step process of DDI by applying triangulation. Such an approach involves multiple analysis methods, thereby providing more robust evidence on the seven-step process of DDI. Therefore, we also contribute to the DDI literature. Finally, this study makes methodological contributions by adopting multiple methods consisting of systematic literature review, thematic analysis and semi-structured interviews. Combination of multiple methods confirms reliability and robustness of the research process.

## Theoretical framework

### Dynamic capabilities theory

The dynamic capability theory (DCT) suggests that the organizations build, integrate, reconfigure their internal and external resources and capabilities to effectively innovate its operational procedure or market offering with a view systematically innovates its way of doing things and adapt to changes in the marketplace (Ambrosini et al., 2009; Teece, 2007; Zollo & Winter, 2002). DCT has been developed as an extension to resource-based view (RBV) to explain the underlying reasons and process how firms can be more innovative in developing competitive advantage in response to the rapid changes in the business market (Eisenhardt & Martin, 2000; Kumar et al., 2016; Teece et al., 1997). According to RBV, firm resources could be categorized as managerial, input-based, transformational and output-based (Menguc & Auh, 2006). As an extension of the RBV, DCT posits that organizations continuously develop new resources and/or new ways of utilizing existing resources to achieve new and innovative forms of a competitive advantage given their resources, path dependencies, and market positions (Teece et al., 1997).

The key to DCT is that it enables the organization to innovate constantly in environmental uncertainties, which are regarded as an important factor in the current age of data-driven technology. In the data-driven digital environment, BDA is regarded as one of the most valuable resources which will be key to design any innovation which has implications for the firm’s competitive advantage (Kumar et al., 2016). To facilitate innovation, DC, eventually, fosters new thinking within the firm by combining skills, data, technologies and expertise to create revenue-producing products and services (Braganza et al., 2017). The firm’s DC is enhanced when the firm uses novel consumer insight extracted from Big Data to understand and meet customers’ unmet needs. Manufacturing firms that are operating in a dynamic big data environment need to focus on developing strong analytics capabilities with a view to adapting and innovating with market and technology developments (Teece, 2014). In BDA research, scholars have most commonly used RBV (Aydiner et al., 2019), DCT (Akter et al., 2016; Chen et al., 2015), organizational information processing theory (Srinivasan & Swink, 2018) in achieving competitive advantage and performance. In a recent study, Dubey et al. (2019) have integrated three theoretical perspectives such as RBV, institutional theory and organizational culture to examine the relationship between institutional factors and resources of the firm and operational performance.

### Institutional theory

The core of the Institutional theory explains changes in the organization while emphasizing the role of the institutional environment in shaping the actions of the organization (Tina Dacin et al., 2002). About the adoption of innovation, institutional theory highlights the effect of external environmental factors and the organization’s capability and organizational goals (Oliver, 1997). As a result, firms in the same industry, for example in a manufacturing context, have to imitate the actions of industry leaders in designing and adopting innovation to survive and succeed (Sun et al., 2018). According to this theory, also as evident in prior literature, pressures from stakeholders can potentially influence the organization’s decision to adopt big data which have implications for innovation (Dubey et al., 2019). Moreover, institutional theory advocates micro-sociological approaches which emphasize on internal dynamic capabilities of the organization. There exists a strong connection between institutional theory, DCT and RBV as institutional theory assumes that external environmental factors have exerted significant influence on the organizations whereas, as per RBV and DCT, organizations try to develop their resources and capabilities to optimize the available economic opportunities. Dubey et al. (2019) argued that institutional pressures affect positively firm resources, which further help the organization’s to build BDA capability to innovate.

## Technology–organization–environment framework

The technology–organization–environment framework (TOE) would be another key factor to explain the theoretical context of this study. TOE suggests the process by which the organization’s innovation initiative is influenced by the technology, organization and environment contexts (Tornatzky et al., 1990). The technology context comprises of necessary equipment and processes and their related internal and external technological capabilities and resources such as BDA, which have significant influence over the technological innovation itself. The organization context refers to the organization’s resources, capabilities and other characteristics, whereas the environmental context encompasses the organization’s macroeconomic context (Sun et al., 2018). For this study, the TOE framework provides the overarching theoretical foundation for our analysis as it complements the DCT and institutional theory.

For this study the dynamic capability theory (DCT) of firms would provide a useful theoretical framework for understanding the sources of firms' strengths and the processes through which firms build, integrate, reconfigure their data-driven resources and capabilities to effectively innovate its operational procedure or market offering (Ambrosini et al., 2009; Teece, 2007; Zollo & Winter, 2002). Institutional theory is key as it considers the environmental factors and organization’s capability and goals in materializing DDI in the context of the manufacturing industry, in line with DCT. And TOE will provide an overarching framework which would emphasize the process, comprised of technology, organization and environment, to facilitate the organization’s innovation initiative. In BDA literature, several studies have adopted a multi-theory approach to explaining a complex process systematically (Dubey et al., 2019; Sun et al., 2018), therefore, we integrated the above-mentioned three approaches, DCT, institutional theory and the TOE framework, to explain organizational adoption and intra-organizational data-driven innovation diffusion more effectively.

## Literature review

### Definition of BDA

The growth of digital technology, the diffusion of smart devices, social network platforms, and cloud computing, and other growing trends such as the Internet of Things and Internet of People have played a key role as data generators (Trabucchi & Buganza, 2019). As a result, firms have to deal with huge sets of unstructured data along with structured data. These unstructured set of big data are being generated because of the users’ online activities that include web navigation, clicks, likes, links, blogging, voices; whereas, structured data are generated through the users’ demographic variables such as name, age, gender, date of birth, address, and preferences. One of the biggest challenges in the BDA environment is to deal with both types of data in order to generate meaningful insights (Akter & Wamba, 2016).

In the extant literature, BDA is generally defined as the vast and complex set of data that require specialized management tools to capture, store, manage and process efficiently (Hu et al., 2014; Madden, 2012; Manyika, 2011). Although the definition hardly does impose any restriction on the data size in terms of any quantifiable metrics, because of the technological advancement, it incorporates a dynamic aspect about the size and length of the data-set to qualify as big data (Lamba & Singh, 2017). While defining BDA, some authors emphasized the variety of data sources; whereas others stressed on storage and analysis requirements of dealing with big data (Akter & Wamba, 2016). In the absence of any concrete conceptual definition, researcher have attempted to define BDA from various perspectives which resulted in that the concept has incorporated various dimensions which include greater scope and volume of information; new category of data and analysis; real-time information; data from non-traditional forms such as social media; new technology-driven data; the latest buzz word (Schroeck et al., 2012).

Akter and Wamba (2016) argued that the academicians and practitioners widely view that big data can be comprehended according to four Vs—Volume, Velocity, Variety and Value. These dimensions were later elaborated to six—Vs, including veracity, value, and variability (Gandomi & Haider, 2015). The volume attribute represents the vast magnitude of data that the firms collect from dynamic, heterogeneous, and ubiquitous resources and devices. These diverse sources and devices enable the collection of ‘varieties’ of data which are structured, semi-structured or unstructured in nature (Gandomi & Haider, 2015). Velocity refers to the speed at which data are generated, potentially analysed and acted upon for further application (Akter et al., 2019). The value of big data refers to its economic value that can be extracted from structured and unstructured data. Variability refers to the variation in the speed of data flow which is caused by the inconsistent velocity of big data (Gandomi & Haider, 2015). Veracity refers to uncertainty and unreliability in the data set caused by the complex, inconsistent and anonymous nature of the data.

### BDA and data driven innovation

Data-driven innovation (DDI) is regarded as an emerging approach by the academics and professionals, to enhance innovation in companies by acquiring, analyzing and acting upon consumer data (Riikkinen et al., 2018; Rindfleisch et al. 2017). DDI refers to the innovation process that adopts techniques and technologies for processing and analysing big data using data-based decision processes. In this case, data plays a key role to the firm’s innovation activities by providing knowledge about manufacturing and operational processes, customers, human capital and technology significant to enterprise (Bharadwaj & Noble, 2015; McAfee et al., 2012). DDI relies on the context of knowledge-based capital associated with digital information, innovative capacity and economic aspects (OECD, 2015). This is quite evident that the analysis of big data provides insights to generate valuable knowledge which influences the performance of the organization by providing a competitive advantage in an enterprise through new ways of productivity, growth, innovation and consumer surplus (Manyika, 2011; McAfee et al., 2012; OECD, 2015). Organizations which have been most successful with analytics capitalized on analytics to create a competitive advantage and innovative ideas or marketing offer. For these organizations, data is a core asset generated from different sources such as customers, partners, suppliers and even competitors (Kiron et al., 2014).

Recent research findings have demonstrated the implication of BDA on different kinds of innovations, from process to product, from architecture to a modular base (Caputo et al., 2016; George & Lin, 2017; Ransbotham & Kiron, 2017; Trabucchi & Buganza, 2019). BDA can not only facilitate an open innovation strategy to explore various business opportunities but also expand the operation of the company by pursuing business model innovation, customer centricity, data orientation management and the implementation of agile practices (Del Vecchio et al., 2018; Trabucchi et al., 2018; Troilo et al., 2017). Findings from Trabucchi and Buganza (2019) suggest that companies can leverage the opportunities provided by the BDA and the pervasive diffusion of sensors to gather a specific kind of data and therefore to design an entire system and business model around the need of data. Rindfleisch et al. (2017) argued that DDI enables the companies to exploit their value and enable a particular kind of product innovation enabled by the chance to gather and analyze data. Companies, such as General Electric, Google, IBM, Airbnb, and Uber, Bridgestone and Nedbank Group Ltd, use strong analytics capabilities to carry out DDIs not only for existing operations but also in developing new processes, products, services, and entire business models.

Despite the positive implication of BDA for innovation, many firms failed to capitalize the idea on how they can leverage big data as a capability to generate innovation success in dynamic marketplaces (Johnson et al., 2017). Apart from infrastructural issues, unavailability of skilled people is another significant obstacle to adopt a data-driven approach through BDA. People, high levels of technical skills in statistics and IT play a key role to use and exploit these systems and to get valuable insights from big data (Kopanakis et al., 2016).

### BDA in manufacturing

BDA is one of the recent technological advances which have strong applicability for DDI in almost every industry, including manufacturing (Babiceanu & Seker, 2016; Moktadir et al., 2019). Data-driven revolution promises to transform conventional manufacturing facilities into highly optimised smart manufacturing facilities that generate intelligence from a big scale of real-time data. These data support help the management to make an accurate and timely decision for the smooth operation of the company (O’Donovan et al., 2015). In modern-day manufacturing, BDA provides the critical technical support to acquire, store, manage and analyze data which help the organizations in demand forecasting, quality control, scheduling and maintenance (Bi & Cochran, 2014; Lamba & Singh, 2017). In the manufacturing sector, BDA has implications in various stages of the product’s life-cycle, design innovation, manufacturing intelligence, cost reduction, quality, efficiency, and making effective promotion strategies (Li et al., 2015). However, as suggested by Dubey et al. (2019) and Moktadir et al. (2019), DDI has become more evident in the context of supply chain management in the manufacturing sector.

There are several leading manufacturing companies which have improved their production processes and enhanced operational performance through BDA (Yadegaridehkordi et al., 2018). Rolls-Royce carried out DDI to produce a higher gear with the Engine Health Monitoring Unit (EHMU) that collects data by the use of specific sensors (Zhong et al., 2016). Wahoo Fitness, based in Atlanta, Georgia, relies on analytics to design innovative sports and fitness products, including workout apps, smartphone-connected fitness devices, heart rate monitors, indoor smart-bike trainers, and GPS bike computers. Gore, a manufacturer of advanced materials based in Newark, Delaware, applied a data-driven research process to design its Gore-Tex waterproof fabric (Ransbotham & Kiron, 2017). Raytheon Corp, a major U.S. manufacturer of weapons and military and commercial electronics has capitalised BDA to establish smart innovative manufacturing plants Although there are several examples of manufacturers’ adoption of BDA in designing innovation; however, the application of BDA in DDI is still in nascent stage in the manufacturing sector (Yadegaridehkordi et al., 2018). Using insight generated from BDA, large scale manufacturers like Volvo, Toyota and Xerox implemented process innovation, built physical proto-type accurately, forecast component failure in advance, lowered production cost, minimized wastage and improved customer service (Young & Pollard, 2012). Vaccine manufacturing firm Merck applied BDA technique to crunch huge amounts of data to innovate the production process to produce vaccines faster and reduce the discard rates (Henschen, 2014). The use of BDA may reveal unexpected insights and further opportunities to enhance performance for the manufacturing operations that are considered best in class. An established European maker of functional and specialty chemicals for a number of industries, including paper, detergents, and metalworking achieved unexpected insights when the company applied advanced BDA techniqued such as neural-network techniques. The chemical company was able to reduce its waste of raw materials by 20% and its energy costs by around 15%, thereby improving overall yield. Similarly, a top-five biopharmaceuticals company applied BDA to significantly increase its vaccine production while incurring no additional capital expenditures (Auschitzky et al., 2014).

In case of facilitating innovation, in the context of manufacturing, internet of things (IoT) and BDA are connected to develop advanced manufacturing information system that not only generate an unprecedented level of data but also streamline the probable impediments and improve forecasting systems (Bi & Cochran, 2014; O’Donovan et al., 2015). The core technologies of IoT such as RFID, Wireless Sensor networks, cloud computing play the key role (Yang et al., 2016). However from the manufacturing company’s point of view, the manufacturing facilities must be able to manage the demands of exponential increase in data production as there are many barriers to the adoption of BDA in manufacturing supply chains such as lack of infrastructure, complexity of data integration, data privacy, lack of availability of BDA tools and high cost of investment. The company must establish the infrastructure, recruit technical people and possess required analytical techniques to extract meaning from these large datasets (Moktadir et al., 2019; O’Donovan et al., 2015).

## Methods

In this study, for collecting data, to identify the steps in DDI we adopted multiple methods consisting of systematic literature review, thematic analysis and semi-structured interviews. This enabled the triangulation of thematic analysis findings and 23 semi-structured interviews from the manufacturing sector in the UK to establish DDI steps. Triangulation is a robust method to reduce bias and ensure the validity of research findings through multiple analysis methods. For example, the findings from the thematic analysis were supported by qualitative interviews or people’s voices to capture rich insights and investigate how people interact with a particular setting, such as data-driven innovation in the manufacturing sector (Silverman, 2011; Skinner et al., 2000). Also, a combination of multiple methods establishes reliability and ensures methodological robustness in such research process (Fusch et al., 2018).

According to Schutt (2018, p. 13) “exploratory research seeks to find out how people get along in the setting under question, what meanings they give to their actions, and what issues concern them”—the aim is to understand “what is going on here”. Following the guidelines of Akter and Wamba (2016), Thomas and Leiponen (2016) in big data research and Tranfield et al. (2003) in management research, we first developed a systematic literature review approach to understand DDI. The objective at this stage was to discover and gather logical evidence pertinent to the research question. A robust systematic literature review method is critical to gather empirical and practical evidence regarding DDI (Sivarajah et al., 2017). As part of this process, the study uses the following research protocol in searching and identifying relevant publications from various databases.

First, we identified relevant search strings that consisted of the keywords ‘big data innovation*’ and other related keywords, such as ‘new data product’ and ‘data-driven innovation’ using wildcard symbols. The study focused on big data-driven innovation as a primary objective, and hence, search strings ‘analytics* AND innovation’, and ‘big data analytics* AND innovation’ were found relevant to address the research question. In Davenport and Kudyba’s (2016) proposed data product development approach, some aspects of data-driven innovation such as conceptualisation, data acquisition and refinement, storage and retrieval are inherent. As part of capturing these critical constructs, search strings ‘big data innovation*’ in association with ‘data-driven innovation*’, ‘manufacturing*’ ‘big data analytics*’, and new data product*’ were established.

Applying the above mentioned search strings, the study identified relevant publications by exploring five relevant databases: Business Source Complete (EBSCOhost), Scopus (Elsevier), Web of Science (Thomson Reuters), ABI/Inform Complete (ProQuest) and ScienceDirect (Elsevier). To ensure accuracy in search process, various inclusion and exclusion criteria were followed, such as the inclusion criterion focused on the title, abstract and keywords and the exclusion criterion focused on avoiding various disciplines (e.g., geology, physics or chemistry) which were not relevant to the study or research question. The database search resulted in 3331 hits. After a careful screening of selection criteria and duplicate publications, 91 publications were downloaded and 21 of them were found relevant to the DDI. However, the study explored pertinent other publications to develop a detailed understanding of DDI.

As part of the thematic analysis following the procedures by Braun and Clarke (2006), the study explored the literature on DDI and found seven predominant steps. Thematic analysis is a robust analysis technique to identify recurrent themes running across phenomena under investigation (Braun & Clarke, 2006) in which each theme is considered as a category or construct or dimension (Fereday & Muir-Cochrane, 2006). The findings of our thematic analysis are very much aligned with Davenport and Kudyba’s (2016) proposed data product development approach. In order to embrace further rigour in the thematic analysis, the study used Krippendorff's alpha (or, Kalpha) as a reliability measure (Krippendorff, 2004, 2007). Kalpha value was used to check the inter-rater reliability of the 7 coded DDI steps following the guidelines of Hayes (2011) and De Swert (2012). Each of the 21 articles was coded independently by two academic judges using a 1 to 7 point categorical scale in which 1 = innovation conceptualisation 2 = data acquisition, 3 = refinement, 4 = storage and retrieval, 5 = distribution, 6 = presentation, 7 = market feedback. We estimated a Kalpha value of 0.82 exceeding the threshold of 0.80 to confirm the validity of the thematic analysis. The next section discusses the findings of the review in detail.

A total of 23 interviews were conducted with data led innovation experts, including professionals from various manufacturing industries in the UK, such as aviation, rail, electronics, semiconductor, construction etc. (as detailed in Appendix 1).

Purposive sampling technique as applied for selecting the respondents because of their role and experience as key decision makers and in managerial positions and representation of a wide-range of sectorial areas. To ensure the right criteria of our respondents, all the interviewees were communicated through the researchers’ professional and personal connections. Since we reached theoretical saturation point and due to continuous repetition of themes, we ceased interviewing after 23 respondents (Glaser & Strauss, 2017; Taylor & Bogdan, 1998). Before the interview, the respondents were informed of the details of the research following a two-way, open communication atmosphere. We ensured ethical issues and confidentiality and anonymity of interviewees during the interview data collection process. The data collection and reporting process took due steps to ensure ethical standard, confidentiality and anonymity of interviewees. We undertook face-to-face interviews at convenient times and places confirmed by the respondents, with an assurance of valuing their any opinion. Interview protocol works as an action plan in the studies where the experiential investigation is subjective as it plays a key role in operationalising the research, and data collection (Yin, 2014). In the this study, the interview guide was initially developed based on the themes identified in the literature (Appendix 2); however, during the interview sessions, the researchers had an open mind and applied probing supplementary questions to note down deeper insights. Each interview lasted for 30–45, and the interview transcripts were analyzed using QSR NVivo 11 software to understand the data-driven innovation process.

We applied template analysis to identify and organize the recurrent themes in a meaningful category from the interview transcriptions. In this research, we identified several broad themes such as Technology in manufacturing innovation, Role of BDA in facilitating data driven innovation and Challenges and outcome of BDA in manufacturing system which were subsequently broken down in codes, based on the research objectives, stream of data, key themes in the literature review and theory (Fereday & Muir-Cochrane, 2006). The relevant themese and codes are presented in Appendix 3. We applied triangulation once all the data were collected from a systematic literature review and semi-structured interviews. The process enabled the researchers to reduce bias and ensure the validity, to ensure that both the findings from the systematic literature review and interviews outcomes complement each other while capturing rich insights (Silverman, 2011; Skinner et al., 2000).

## Findings

### Step 1: Conceptualizing innovation

Conceptualizing the innovation and need of the market is regarded as a very crucial stage of DDI as it provides guidelines for the rest of the process. This process of conceptualization should precede the acquisition, refinement, storage and distribution of data and fine-tune with market feedback stage (Davenport & Kudyba, 2016). At this stage, the organization assesses the market to identify a need to satisfy and to envision the innovation along with the available data sources required for that innovation. While assessing the market needs, the organization analyses the customers’ demographic, psychographic and behavioral characteristics, their current consumption pattern, market trends, available technology, competitors’ strategies for innovation and changes in the environment. One of the participants suggested the role of data from the market and stakeholders in conceptualizing the innovation idea.

In our organization, innovation is a continuous process. Having said that, every innovation, whether it is product innovation or process innovation, is initiated with and backed up by market data. We continuously monitor the market (customers, competitors and other stakeholders) to innovate any product. …. in facilitating any of our innovation, market assessment and feasibility study is a key factor since it provides us so many important insights about the current market. This helps us to envision the future trend and trajectory of the market in which our innovation can fit it. (Male, age 42 with 12 years of experience)

To conceptualize customer-centred innovation, the organization facilitates customer engagement to generate insight into the innovation and prototyping processes. Several manufacturers engage the customers through competition, artificial intelligence, telematics and other offline and online services which generate ample data to shape the innovation process. Companies like Bridgestone conceptualize about data-driven innovation in its business process and product development through analytics generated from customer engagement (Ransbotham & Kiron, 2017). While emphasizing the role of the customer in data generation, one participant suggested that,

Although we follow a rigorous process to initiate a ground-up innovation project, we rely heavily on the data generated from the customers, particularly to understand the gap in the customers’ need fulfilment and to know their perception about competitors’ products. Therefore, we engage the customers in generating analytics about their preference, perception, prediction and purchasing pattern which immensely support us to conceptualize innovation ideas. (Male, age 36 with 6 years of experience)

On a different note, large manufacturers have followed a more structured method for incubating an innovation idea. They have a dedicated team who explore opportunities, analyze relevant data to shortlist any actionable idea. While commenting on the structured method, one participant suggested that,

Our innovation activity follows a rather systematic and organized structure which comprises of the large R&D team, extensive research work, the participation of stakeholders. In our organization, we very rarely deal with the consumers as we deal in B2B market where suppliers and other stakeholders also play an important role in the overall innovation process. Therefore, in our organization, the conceptualization process of any innovation is the outcome of another structured procedure, which can be labelled as ‘pre-conceptualization’ where several analyses and research are executed to conceive any innovation idea. (Male, age 40 with eight years of experience)

At this stage, probable ideas of any innovation are generated and scrutinized to address various problems and explore untapped opportunities, following a structured or semi-structured method. The outcome of the assessment of various data will help the management to conceptualize the initial idea, scope and type of innovation which will determine the data and resources to drive the innovation. Moreover, this stage outlines the role of key enablers for market innovation, which shed light on the market need, portfolio fit, and product-capability fit in terms of taking a decision about innovation.

### Step 2: Data acquisition

To facilitate DDI, the generation and acquisition of data would play an instrumental role (Ransbotham & Kiron, 2017). In the industrial manufacturing sector, data acquisition (DAQ) refers to a process that, using computer-based hardware, sensors and application, incorporates and measures physical or electrical phenomena such as noise, visual stimuli, pressure, temperature, voltage. With the advancement in technology and for the emergence of the industrial internet of things (IoT), the demand for industrial data acquisition systems has been facilitated. However, with the advancement in technology, powerful microprocessors are now used to perform complex data processing and represent the data as actionable indicators. Data acquired from these sources, particularly from the machine source, are sent to the enterprise applications (e.g., ERP, EAM systems). While emphasizing the role of hardware, software, artificial intelligence, and technology several participants suggested that,

Most data acquisition scenarios assume high-volume, high-velocity, high-variety, but low-value data, making it important to have an adaptable and time-efficient gathering, filtering, and cleaning algorithms that ensure that only the high-value fragments of the data are actually processed by the data-warehouse analysis. (Male, Age, 55 with 20 years of experience)

Like many other organizations in manufacturing sector, we follow a structured method and have applied several computer-based software and applications such as supervisory control and data acquisition (SCADA) which is a combined system of software and hardware that enables us to acquire, monitor, and process real-time data and later sent to the enterprise applications (e.g., ERP, EAM systems). (Male, Age, 58 with 16 years of experience)

There exists a strong relationship between big data capabilities and Artificial Initiative (AI) initiatives and both of them are influencing each other either way. AI has a significant role in providing the digital framework to acquire data in a large volume (big data). On the other hand, AI needs a large volume of data to develop its machine learning capability. (Male, Age, 51 with 17 years of experience)

The data acquisition and aggregation process should coincide with the organization’s advanced business model and functionalities and be adapted to the elaborative product architecture developed by the firm at the early stage of this model. In the current day of digital technology and big data, there is an explosion of data as people leave enormous footprints through their online activity. From a company’s perspective, data can be acquired generally from unstructured (e.g., social media activities, posts, tweets, blogs, image, and video data), structured (e.g., customers’ account data, retail transactions, and sensor data) and machine sources (wired and wireless smart sensors, cloud computing, crowdsourcing, wearable devices and the IoT) (Yang et al., 2016; Zhu et al., 2017). The participants also underscored the role of algorithms and various devices and IoTs in this regard,

The emergence of IoT has facilitated the data acquisition process in an exponential manner. Data acquisition through IoT is a very complicated and technical process. However, as the project manager, anyone can have the liberty to be little ignorant about the technical side and focus mostly on the how the process works and to interpret productively the conversations with the whole engineering process.” (Male, Age, 49 with 11 years of experience)

In the pharmaceutical industry, predictive analytics algorithm can help in identifying demographic factors to observe the incidence of diseases, their progression and outcomes…..…enabling to identify predictive biomolecular signatures of response to vaccination, vaccination will shift from the classical “one-size-fits-all” paradigm to a personalized approach and similarly, personalised medicine for individual. (Male, age 41 with 8 years of experience)

Many companies acquire data through the data sharing process. German automakers *BMW, Daimler,* and *Volkswagen* formed an alliance and acquired Berlin-based digital mapping company *Here* with a view to creating a crowdsourcing service. All the automakers were benefited from this company as it captured real-time data from drivers about detailed road conditions on a single platform (Ransbotham & Kiron, 2017). One of the participants also highlighted this point.

…….and sometimes, we need specialized data which we procure from third party suppliers. Like many other companies in the electronic and automobile industry, we acquire data through data sharing services, which save effort and budget for such quality data. Furthermore, through professional market research agencies, we collect a lot of market data about the customers and competitors. (Male, Age, 58 with 16 years of experience)

For the DDI, an abundance of data is a key factor to initiate and facilitate the process. Companies acquire data from multiple structured and unstructured sources, including IoT and artificial intelligence. Some organizations even outsource the data acquisition process and share the resources with others. However, to be most effective about data acquisition and generation, data governance needs to be embedded in an organization’s culture.

### Step 3: Refinement

Data refinement refers to a complex process of converting unstructured, uncategorized and unrefined sets of abstract data and eliminating data volatility and redundancy into the implementable structure or usable insights. Although Meyer and Zack’s data refinement process remains quite relevant; however, Davenport and Kudyba (2016) argued that the data refinement process should rely on advanced analytic methods while facilitating new data sources. Rindfleisch et al. (2017) redefined data refinement as innovation from data (IFD) which applies digital tools to acquire and analyze consumer data to enhance innovation by obtaining insight derived from digital data observing consumers’ online behavior. For this purpose, firms require advanced modelling software and skilled people in a data-rich environment. While explaining the process of data refinement, our participants commented that,

The challenge is to make sense of the data, reveal the patterns in it and use them for operational improvements and to support strategic decision making. Advanced modelling software, data mining tools and skilled people are the key factors in refining the large scaled data. Software programmes offer lots of modelling and visual presentation of data; however, down the line, it is the people’s technical and analytical skill and capability that is more instrumental in refining data. (Male, age 35 with 6 years of Experience)

Refinement of data is very vital to process and extract the crucial information/ data in a structured way so that it could be readily analysed. However, it is very important to ensure that data do not lose validity and accuracy in this process. It is advisable to include some technical features in BDA so that they could take into accounts the error models and well-recognised constraints. Cloud technology could incorporate these features while storing and refining them. (Male, age 41 with 8 years of experience)

……..it is utmost important for the managers that they have to take into account huge volume of available data which they have to optimise for their activities. A number of intelligent techniques including fuzzy logic, neural networks, genetic algorithms, data fusion, clustering have evolved to be well-suited for textile manufacturing industry since they can effectively deal with huge data considering diverse and complex correlations and dependencies between them and uncertainties related to sensory quality attributes, consumer behaviour, manufacturer’s knowledge, and so on. (Male, age 50 with 15 years of experience)

The data refinement and IFD process are significant because for its innovation process, firms actively acquire and analyze data from consumers who are largely passive data providers (Bharadwaj & Noble, 2015). This stage of the data management process bears sheer significance as the application of modelling tools extract out the insights which are instrumental for the rest of the process. The outcome of data refinement also helps the firm to redefine the process of innovation and understand customer perceptions. Moreover, the manufacturers can utilize the insights to reengineer their operational process and enhance supply chain efficiency. Walmart is actively seeking to build trust and loyalty through its data refinement by developing a program called One Version of Truth. Having partnered with the data firm Nielsen, Walmart ensured a smooth way for its manufacturers, vendors and supply chain partners for using its data to provide a consistent view of the market so they can leap on opportunities, reduce costs and also improve the shopper experience (Pearson, 2017).

In large corporations like ours, management people have constant access to large volumes and sources of data which can feed Artificial Intelligence (AI) algorithms to refine and organize them, to detect patterns and trends and to interpret stakeholders’ perception and behaviors. Rather than only relying on some subsets of data for analyses, we need to integrate large volume of data, AI algorithms, and robust hardware and programmes to generate several outcomes. (Male, age 42 with 12 years of Experience)

Related to the big data management, the organization should not focus only on the data acquisition process rather it should develop and implement a comprehensive data strategy which would guide about the data collection (acquisition), volume of data, data refinement and processing. Guidance about data refinement is important as it would suggest about various issues of data management such as what type of, when, how and from where to collect and eventually how to interpret. (Male, age 51 with 17 years of experience)

### Step 4: Storage and retrieval

Storage and retrieval have become increasingly important in the emerging big data ecosystem. They provide the platforms to ascertain the variety, velocity, and volume of un-purified and unpolished data, which in turn help to manage big data in an efficient manner. Business value of data innovation needs to be appreciated for determining the storage strategy and planning. This might include compliance needs, data protection, removal of redundancy, access and response. Retrieval of big data takes in account of advancements in query and search processing capabilities with the use of algorithms to facilitate the raw data. Many organizations perform the acquisition and storage of data in rather unstructured formats and process them over time. As mentioned by a few participants,

It is pivotal to gather and store data into a database which is accessible and can be modified, combined, and furnished where appropriate. Otherwise, data become unproductive.” (Male, age 41 with eight years of experience)

Large manufacturers have developed a hybrid storage model for BDA which have been able to meet the scalability demands and have support for BDA solutions such as Hadoop, Cassandra etc. Generally, a storage architecture comprises of direct attached storage (DAS) pools and clustered network attached storage (NAS). Over time, object storage has evolved as it could incorporate high capacity files even over billions. An ideal storage system should ensure strong security measures with full control, facilitate migration to a cloud system and provide easy retrieval. (Male, age 51 with 17 years of experience)

There are many different areas of supply chain management where previously stored data can be retrieved for further applications. Such as, warehousing and transportation are both areas where Big Data tools can be used with great Return on Investment. (Male, age 50 with 25 years of experience)

Cloud offers a powerful architecture to provide data storage, retrieval, and processing for a given organization, which integrates parallel data processing frameworks to access both cloud resources and execute programs (Gunarathne et al., 2013). Cloud computing, which comprises of various resources, i.e., end-user applications, personal data or Database Management Systems (DBMS) are provided by a third party over the internet like services. It provides opportunities for organizations to be flexible in their technology platforms and simplifies big data analytics (Davenport & Kudyba, 2016; Hashem et al., 2015). However, although cloud computing has received enormous attention, it carries risk related to data security and privacy since the data is stored in a scattered manner in different devices in different locations around the world. Therefore, many organizations are still reluctant to adopt this technology despite its advantages such as flexibility, efficiency, and scalability (Usmani et al., 2018). According to a participant

Amongst the storage system of large scale data, the latest technology is cloud computing, which is omnipresent in almost all the large organizations. This technology brings in various advantages such as huge capacity, scalability, efficiency and flexibility; however, we must not overlook the potential threat it carries in terms of security of data since the data are stored in a scattered way on various machines in various geographical areas. Therefore, we need to ensure a reliable and secured cloud environment to store these BDA. (Male, age 40 with 12 years of experience)

While considerable effort has been made to highlight the applications of big data from a service point of view, little is known about the importance of storage and retrieval BDA from the manufacturing point of view. Prior advancement on ICT has increased the efficiency of data collection, storage and retrieval of publicly accessible secondary data by various innovations, such as cloud storage (Akter et al., 2019). This study puts forward the importance of efficient storage and retrieval mechanism of big data by different business units, including the manufacturing sector.

### Step 5: Distribution

In today's environment, the success of an organization is dependent on data innovation, data structure and data product life-cycle. With the advent of the Web, the distribution options of data products have been revolutionized. Near-real-time availability, access and updating of data products offer an expansion of business globally for many organizations. However, Web access by traditional computers has been superseded the evolution of mobile devices including smartphones and tablets etc. (Erraissi et al., 2017). This has necessitated the modification of format and designs of information products to suit the need of users. The participants pointed out that,

…. the distribution policy of BDA of an organization should ensure the availability of relevant data for decision making. Authorized users receive access to the central database (hardware and cloud-based) to generate and analyze real-time data about customers’ behavioral pattern, product’s usage pattern, and innovative design. Of these data, customers’ behavioral data should be made available in an encrypted way to comply with the general data protection regulations. (Male, age 48, with 13 years of experience)

The big datasets generally go through initial diagnostic, analytical techniques to generate descriptive and predictive findings before being available to the authorized personnel. The original datasets are also made available to various teams (distributed) for further analyses such as content analysis, time series modelling, geographical location clustering, text and/or visual content processing. However, the overall distribution and analyses process of data could be a complicated matter depending on the organization’s data strategy in terms of the volume of data and data storage. (Male, age 51, with 17 years of experience)

Cloud computing has concurrently added a new dimension in order to leverage the scalable and cost-effective solution in big data analytics. It has enabled the organizations to gather the benefits by responding to users' expectations and needs as well as alleviating complexity in operation. For example, multinational companies use distribution channels to manage inventory in real-time so that they can connect retailers globally with synchronized data warehouses and manage commercial transactions in a completely automated fashion (Davenport & Kudyba, 2016). Moreover, timing and frequency remain critical for distribution of data products in the digital economy so that it could take care of issues related to access and availability. Importance of data availability was highlighted by the participants-

Timing and frequency of data distribution is very important in a sense that required data are available at a right time and in right amount”. (Male, age 50 with 25 years of experience)

It can aid in running efficient clinical trials, for real time monitoring data. (Male, age 41, with 8 years of experience)

Access to the real time data is very important for decision making at every stage of management and operations of innovation. Therefore, to us, (the professionals in the industry) the stored data should have been distributed and available at real-time or near real-time speed. To deal with the large volume of data, many organizations use Hadoop Distributed File System (HDFS) to organize and distribute data across parallel computing clusters. (Male, age 58, with 16 years of experience)

Most of the organizations distribute the data after refining and conducting analyses. Before the data analysis, data are to be prepared through data mining, cleansing, aggregation and integration, which refers to extracting and cleaning diverse, vibrant, divergent, and interrelated data (Chen et al., 2013; Sivarajah et al., 2017). There are several analytical methods available for analyzing big data such as Topic Modeling, MDS, K-Nearest Neighbors (k-NN) Clustering with data visualization using free online content for automated competitor analysis, text mining, sentiment analysis and comparative analytics, algorithm analysis and machine learning. From a taxonomical standpoint, most scholars clarify BDA techniques as descriptive, predictive and prescriptive (Delen & Demirkan, 2013; Wang et al., 2016) and inquisitive, pre-emptive analytics, and diagnostic analytics (Sivarajah et al., 2017; Wedel & Kannan, 2016). However, organizations should identify relevant methods which are suitable for the problem at hand, and develop people skill (Akter et al., 2019).

To provide the business value of BDA, it is very important to distribute the data and findings to support specific as well as unspecific organisational units. In fact, the efficiency in distributional mechanism can play crucial role for searching and utilisation of existing BDA based knowledge across different functional units including the manufacturing department as contented by Mikalef et al. (2018: 571) “while some studies assume that leveraging BDA capabilities is sufficient to provide business value, it is important to examine the mechanisms of inertia that act in inhibiting their value.

### Step 6: Presentation

In Big data analytics, context plays an important role in the presentation or application stage of information product. In this step, the performance of each of the preceding value-added steps is scrutinized– for instance, it evaluates whether the user possesses appropriate context to make use of the content and information products corresponds to a distinctive segment of the market (Meyer & Zack, 1996). The user interface plays a significant role in the intended use of products-the simplicity of the information products to use adds more value in terms of payoff. However, analytics are preferred over simple data provision in the digital economy with some important constants.

Data should be readable and easily resolved by analytical methods and processes to facilitate query, modelling and subsequent analysis. In the data-driven innovation process, Big Data could be unreliable, dynamic and diverse in nature, which necessitates that it should be readily accessible and integrated for the purpose of data analysis and mining. (Male, age 41 with 8 years of experience)

DDI in the manufacturing sector will be successful if the presentation of data is effectively done. Properly presented data will improve the overall DDI in manufacturing. (Male, age 40, with 12 years of experience)

An increasing number of organizations are using or attempting to use an agile manufacturing system that enables the manufacturing unit to respond quickly to customers’ expectations and relevant market changes. Well-presented BDA can significantly contribute to controlling cost and maintaining quality by highlighting the changes through strategic information. While simple information products were appropriate for the majority of the users, more advanced and cutting-edge analytics-based products such as forecasts, predictions, and probabilities provide organizations differentiation and competitive advantages over others (Davenport & Kudyba, 2016). The users of the data must be able to visualize the outcome of the raw data, which is a key factor for presenting the findings out of the large volume of data. If the executives have access to the voluminous latest data, it will benefit the organization which has the means and methods to process, analyse and present this. Presenting and visualization of data is important as it helps to interpret the patterns and trends that are present in the data. This has resulted in manufacturing companies investing a substantial amount in developing artificial intelligence and machine learning (Seddon & Currie, 2017). The importance of effective data presentation was stated by the participants-

Collecting large chunks of big data is worthless if we fail to harness the insights lying underneath for which visualization of data is the key. There are many visualization tools available which work standalone or be integrated with another application. Established and big names in the industry use various updated software and applications such as D3, Datawrapper, Powerbi, Oracle Visual Analyze, Qlikview, Google chart etc. Every company prefers the tool that works best for them to convert the raw data into insight and identify the trends and patterns in order to make grounded decisions. (Male, age 48, with 13 years of experience)

### Step 7: Market Feedback

A mechanism for exploiting user feedback and information extraction process from market place empowers data-driven innovation industries to gain customer insights, informs the competitive nature of data products. The requirement for innovation and monitoring product usage is usually supported by the availability of new data sources and maintaining judicious assessment (Leibowitz et al., 2012). In the analytics-based product development process, this step is consistent with a “lean start-up” context which puts emphasis on periodic refinement of the data products over time to support continuous innovation (Davenport & Kudyba, 2016). The connection between customer feedback and innovation was mentioned by a participant-

Customer feedback is important in case of DDI because this improves product and services, helps to measure customer satisfaction, creates a best customer experience, provides data for taking business decisions, becomes a source of information for other consumers and keeps customers. (Female, age 44 with 13 years of experience)

User feedback is acquired in market research and social media platforms can be effective in facilitating a feedback and information extraction process from the marketplace (Callegaro & Yang, 2018). Many organizations monitor and catalog direct comments on social networks in addition to online polls and surveys to appreciate user satisfaction and needs. With A/B or multivariate online testing approaches, any online information product can be assessed in ease of time. Besides flash surveys, interactive blogs are capable of leveraging user impressions about existing information products. Bootstrapping Usability Test can be employed to simulate a real usage of an information product and acquire unbiased feedback, which can then be analyzed to enhance products continuously. At the same time, communicating user and analyzing digital metrics on product use, i.e. views, clicks, downloads, and bounces can facilitate continual innovation in a data-driven industry (Davenport & Kudyba, 2016). This was explained from a particular industry context by one of the participants-

We put a lot of emphasis on constructive feedback from every concerned stakeholder before the commercialization of our innovation. Even after the launch of the product/service we put sincere importance on the customer feedback and strive to make continuous improvement of our innovation. We collect the market feedback following a reliable and comprehensive method which comprises of offline, online, in-person and real-time feedback as it enables us to receive large amount of data. (Male, age 39 with 11 years of experience)

BDA assist fashion industry particularly with the market feedback which generates most value for the manufacturing process as it provides insight and assists decision making or information in the form of patterns. (Female, age 44 with 13 years of experience)

Although the strong association between BDA and market feedback is clear, it is important to differentiate between selling and manufacturing strategies. As such a manufacturing firm can be more resilient to respond to the market feedback is combining two different units of the organisation (e.g., marketing and production). Therefore, the assumption that the market feedback is mainly related to marketing division is misleading, and it is significantly linked with the manufacturing firms/unit.

## Discussion

The findings of this study have several theoretical implications, particularly for the manufacturing firms to consider data as a vital resource to design and implement DDI. Very few studies have investigated the role of BDA in data-driven innovation, particularly, in the context of manufacturing sectors. In the extant literature, there are several studies which have explored and examined the role of BDA in case of the manufacturing sector. However, the role of BDA in facilitating innovation in case of manufacturing industries have received very limited research attention although several scholars have highlighted the importance of adopting BDA for enhancing the performance of manufacturing firms while enhancing their competitive advantage (Dubey et al., 2019; Sun et al., 2018). In this study, we have adapted the seven-step process for data-driven innovation (Davenport & Kudyba, 2016; Meyer & Zack, 1996) and explored empirically how BDA would play a key role in the context of the manufacturing sector. The findings of this study would contribute to the literature of BDA and DDI.

In literature, there are several instances to emphasize the innovation process. To conceptualize and materialize an innovation process, data plays the most important role since most of the leading organizations rely on data to create, refine, and generate innovative output (Davenport & Kudyba, 2016). The original process, suggested by Meyer and Zack (1996), addressed the issue from the viewpoint of information product development in which raw data sources provide inputs to the process of producing a product. Davenport and Kudyba (2016) adapted and moderated the process in consideration of the evolution of various technologies and data sources in today’s digital economy, particularly big data. Our findings have elaborated the framework in light of the empirical findings in every step of the process while isolating the significance of data analysis, which was not suggested in the original seven-step framework.

Data is regarded as the next most important resources to develop and implement any strategy. To ensure its proper application and avoid misuse and an unscrupulous business venture, several regulations are being applied. In the UK, with the GDPR in practice, the manufacturing companies have to be very careful in dealing with the source and identification of data. This has influenced significantly the data collection, storage analysis and distribution process. Our findings suggest that most of the organizations conduct preliminary data analysis while storing the data. The final analysis carried out while distributing and presenting the data and strict regulations are being followed throughout the whole process. Depending on the organizational requirement, BDA analysis techniques could be descriptive, predictive and prescriptive, inquisitive, pre-emptive analytics, and diagnostics.

The findings of the study also highlighted the role of multiple stakeholders for DDI in the manufacturing context. Throughout the DDI process, customers are regarded as the primary sources of data; however, in a manufacturing context, organizations have to consider other stakeholders such as suppliers, retailers, strategic partners as important as the consumers for generating data to conceptualize any innovation idea.

Our findings suggest that to administer high-volume, high-velocity, high-variety datasets, the entire data management process is now completely machine-dependant due to the widespread availability of cloud computing, smart devices, artificial intelligence, internet of things, machine learning and deep learning. Considering the severity of competition, an abundance of data and strict regulations, the organizations need to develop and implement a comprehensive data strategy which would guide every step of the DDI framework, starting from acquisition, refinement, analyzing, distribution and presenting.

**Fig. 1:**
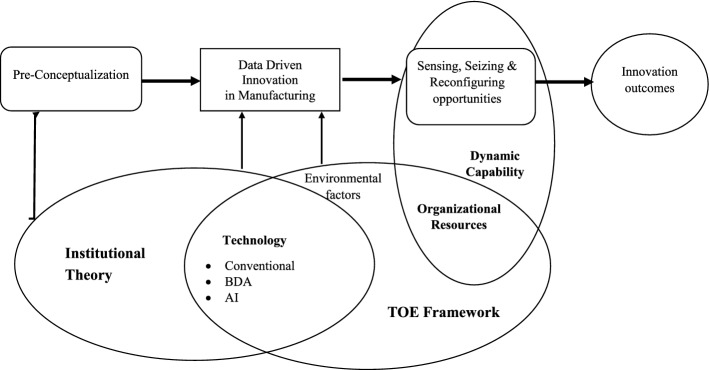
Conceptual framework of how DDI

The results of this study supplement the emerging and existing literature on big data analytics capability of the firms (Akter et al., 2016; Dubey et al., 2019; Sun et al., 2018; Trabucchi & Buganza, 2019). The role of DCT, institutional theory and the TOE framework for the adoption of technological innovations is well discussed in the literature (Dubey et al., 2019; Zhang & Dhaliwal, 2009). The findings highlight the fundamental role of BDA in designing and implementing DDI in manufacturing firms which bears immense significance for the industry to streamline the organization’s data management procedure to sense, seize and reconfigure its resources with a view to materialize innovation. Our findings also established that a manufacturing organization’s capabilities and digital resources are key to drive innovation initiatives. Organizations need to strive such innovation constantly as a result of several stakeholder and institutional factors. Thus our findings have enhanced the theoretical application of DCT and institutional theory. Moreover, the findings enhance the overall TOE framework as it facilitates the DDI through technology, organization and environment factors. Another key theoretical implication of this study is that it has incorporated multi-theory approach to explain organizational adoption of data as resources and intra-organizational data-driven innovation diffusion more effectively. In BDA literature, very few studies have adopted a multi-theory approach, which should be an important consideration, in light of the complex nature of the digital business environment. In extant literature there are several studies which have adopted multiple theories as it provides the researchers to explore various contexts and opportunities of enlarged understanding of the phenomenon (Dubey et al., 2019; Li et al., 2020; Sun et al., 2018; Turunen & Finne, 2014; Zhou, 2011). Moreover, the use of multiple theories and frameworks provide a comprehensive view on the difficult and novel research issue to understand the problem and formulate the conceptual framework. The study of innovation is a complex issue and the present-day research problems are injecting various topics which are confounding the area of innovation. Therefore, to address such circumstances, adoption of multiple theories and framework remain suitable (Dalglish & Newton, 2002; Wolfe, 1994). In this study, the multiple theoretical lenses draw upon a rich set of established theories and framework such as DCT, Institutional theory and TOE framework which are interrelated and synergistic, as a result, they have enabled the authors to develop a more theoretically inclusive analysis and a comprehensive understanding of resource integration and utilization in achieving innovation (Salonen & Jaakkola, 2015). Institutional theory, TEO and DCT are connected with each other and provide a comprehensive theoretical framework to understand innovation in the manufacturing sector as a result of adoption cutting age technologies. This research has approached the complex topic of innovation which has become more complicated with the involvement of DDI and other technologies. The adoption of Institutional theory, TEO and DCT enabled us to explore the organizational adoption and intra-organizational data-driven innovation diffusion more effectively. Institutional theory plays the key role in consolidating the environmental factors and organization’s capability and goals to implement DDI in the context of the manufacturing industry. DCT suggests that organizations develop their resources and capabilities to optimize the available economic opportunities. And TOE create the all-encompassing framework and technology, would emphasize the process, to facilitate the organization’s innovation initiative. The findings and the theoretical framework are summarized in Fig. 1.

The practical implications of the findings from the present investigation in regard to big data analytics and data-driven innovation are to provide meaningful insight into the scopes, opportunities and challenges for implementation in manufacturing industries. Firstly, they can help policymakers to identify what type of skills are effective in terms of data analytics. There are a number of points that are important for the policymakers particularly the importance of skill development. The findings of this study emphasise the important role of skilled people for the best result from big data analytics in general and data refinement (Step 3) in particular. Secondly, they can assist managers of manufacturing firms to be more competitive by the applications of big data. Overall findings will ultimately help their ability to understand the process of big data collection, processing, analysis and interpretation in greater depth and provide the key to understanding a phenomenon and validly interpreting as well as managing the feedback for the manufacturing processes. The findings will help managers and business owners in devising strategies to use big data analytics to understand their business better and improve their decision making more efficiently. The findings have also provided the basis on a critical view of the new tools and opportunities to advance the science of measuring and influencing human thoughts, emotions, and actions to drive innovation. The findings show that people and technology are complementary resources and bring the best result when support each other to implement three key steps—big data collection, processing, analysis (Mikalef et al., 2018).

## Conclusions

The findings of our study confirm that manufacturing organisations with robust analytical capabilities can use big data not only for exploiting the existing operations but also for exploring new products and business models. The findings also highlight the critical steps of how big data are being used in manufacturing from conceptualisation to commercialisation of data products. Throughout the process of innovation, stakeholder involvement and the dynamic analytical capability are critical to address the intellectual, marketing and legal issues, such as data privacy, ownership and use. The findings also reveal that manufacturing firms with strong data governance culture enable data sharing within and across the industry, which facilitates the innovation process. Overall, in the manufacturing, big data-driven AI and machine learning have emerged as an exciting platform to accelerate innovation, value and productivity.

## Limitations

This study has several limitations. First, the study was conducted in the UK context. DDI in manufacturing can vary context to context. As such, future studies should take into account the role of situational variables in generalizing the findings to a new context. However, the proposed DDI process in the manufacturing sector, i.e., conceptualizing the innovation, data acquisition, refinement, storage and retrieval, distribution, presentation and market feedback should be similar across the contexts. Future studies with new contextual applications will confirm our results and add new knowledge. Second, this study is only limited to DDI in manufacturing in the UK. Similar explorations with other countries and their differences might reveal interesting findings. Finally, due to the nature of the qualitative cross-sectional study, one-time data collection is based on convenience/judgement sample which may not be representative of the population. We recommend a longitudinal study with a large sample size to validate our findings using other research methods such as surveys/experiments.

## Appendix 1

Sample distribution

**Table:**

	Count (n = 23)		Count (n = 23)
*Age*		*Gender*	
35–40 years	12	Male	19
41–45 years	3	Female	4
46–50 years	5	*Experience*	
Over 50 years	3	Below 6 years	3
*Industry*		6–10 years	8
Construction equipment	2	11–15 years	8
Aerospace	2	Over 15 years	4
Automotive	4	*Education*	
Electronics and electrical	2	Postgraduate	18
Chemical	1	Doctorate	5
Textile	4	*Organizational position*	
Manufacturing consultancy	2	Manager	7
Pharmaceutical	1	Middle management level	11
Information technology	2	Senior Management level	5
Food and beverages	1		
Industrial engineering	2		

## Appendix 2

Interview Guide

1. To what extent are you aware of the applications of BDA in manufacturing industry? How do these BDA work in manufacturing industry? Could you please explain a few in the context of your company?
2. What benefits do you see in the use of BDA in manufacturing industry? Do you think that the benefits of using BDA command the investment in new technologies to adapt current production?
3. Discuss the role of BDA in facilitating data driven innovation
4. Step 1: Conceptualizing the innovation and the process:
  - How does the company assess the market needs and conceptualize the innovation?
  - Discuss the significance of this step for rest of the DDI process
5. Step 2: Data Acquisition:
  - Discuss about various processes and methods of data acquisition for innovation?
6. Step 3: Refinement:
  - Does the refinement process rely on advanced analytic methods and skilled people while facilitating new data sources?
  - What are the probable outcomes of data refinement process (e.g., process innovation and understanding customer perceptions)?
7. What do you think about/do in practice about the storage, distribution and presentation of data that facilitate DDI in manufacturing sector?
  - How do they affect the overall DDI process in manufacturing?
  - How important is the timing and frequency of distribution of data?
8. Step 7: Market Feedback
  - How are the market feedback collected and applied in case of DDI?

## Appendix 3

Relevant themes and codes for data analysis.

**Table:**

Research themes	Theory driven	Data driven
Technology in manufacturing innovation	Manufacturing and operational technologies for innovation	AI, BDA, IoT, computer-based software and applications for managing volume of data, data sharing services
Role of BDA in facilitating data driven innovation	Steps of DDI process: Conceptualization, refinement, application, Feedback	Pre-conceptualization, dynamic capability
Challenges and outcome of BDA in manufacturing system	Infrastructural, Management and Resources	Resources availability, Skilled people, Management Policy, Integration of organization’s view, Automation, Operational Efficiency, innovative outcomes and environmental issues
